# Percutaneous Cryoablation for Renal Cell Carcinoma

**DOI:** 10.15586/jkcvhl.2015.34

**Published:** 2015-06-09

**Authors:** Tsitskari Maria, Christos Georgiades

**Affiliations:** 1 Vascular & Interventional Radiology, American Medical Center, Nicosia, Cyprus; 2 Johns Hopkins University, Baltimore, USA.

## Abstract

Renal cell carcinoma (RCC) is the most common type of kidney cancer in adults. Nephron sparing resection (partial nephrectomy) has been the “gold standard” for the treatment of resectable disease. With the widespread use of cross sectional imaging techniques, more cases of renal cell cancers are detected at an early stage, i.e. stage 1A or 1B. This has provided an impetus for expanding the nephron sparing options and especially, percutaneous ablative techniques. Percutaneous ablation for RCC is now performed as a standard therapeutic nephron-sparing option in patients who are poor candidates for resection or when there is a need to preserve renal function due to comorbid conditions, multiple renal cell carcinomas, and/or heritable renal cancer syndromes. During the last few years, percutaneous cryoablation has been gaining acceptance as a curative treatment option for small renal cancers. Clinical studies to date indicate that cryoablation is a safe and effective therapeutic method with acceptable short and long term outcomes and with a low risk, in the appropriate setting. In addition it seems to offer some advantages over radio frequency ablation (RFA) and other thermal ablation techniques for renal masses.

## Introduction

Renal cell carcinoma (RCC) is the most common type of kidney cancer in adults and the third most common malignancy of the urinary tract. With the advance and increasing use of cross sectional imaging techniques more cases of RCC are detected at an early stage and when they are clinically occult (1). Surgical resection has been considered the standard of care for patients with localized RCC with partial nephrectomy traditionally being the intervention of choice. Despite this, most patients undergo radical nephrectomy, as the availability of physicians able to perform partial nephrectomy has not kept up with demand. The increased incidence of small renal masses resulted in the development of nephron sparing surgical techniques aiming at preserving renal function. Partial nephrectomy when feasible, either open or laparoscopic, is considered now the gold standard treatment for this subgroup of patients (2). Many patients however are poor surgical candidates, due to old age, the presence of comorbidities, multiple tumors, or compromised renal function. All of the above facilitated the introduction of less invasive ablative techniques as an alternative to extirpative surgical management for selected patients. Several studies demonstrated that ablative techniques can achieve effective local tumor control and are associated with less renal parenchymal loss and morbidity than partial nephrectomy (3). The recent American Urological Association (AUA) guidelines for the management of clinical stage T1 renal mass recognize both radiofrequency ablation (RFA) and cryoablation as viable treatment options for selected patients (4). Ablative methods were first widely used for liver lesions. Renal ablation has also been used during open or laparoscopic surgery since 1995 (5).

## Current Literature on Percutaneous Cryoablation

Recently percutaneous ablation techniques have been gaining acceptance and popularity as nephron sparing treatment for low stage RCC, based upon favorable outcomes in initial studies, low incidence of serious complications, less immediate morbidity and mortality compared to surgery, lower cost and faster mobilization (6). Several studies demonstrated that short and intermediate outcome data following percutaneous thermal energy ablation were comparable to surgery, with clinical success of 90% overall and even greater (more than 95%) for tumors smaller than 3cm (7, 8). Psutka et al. reported that RFA can provide a durable long term oncologic outcome in patients with T1 renal cancer. They retrospectively reviewed the long-term oncologic outcome for 185 patients with sporadic T1 RCC and median follow-up of 6.43 yr. The authors reported a 5-yr recurrence free survival (RFS) of 95.2%, 5-yr disease free survival (DFS) of 88.6%, and 5-yr cancer specific survival (CSS) of 99.4%. (9). Oleweny et al. recently reported comparable 5-yr survival rates for RFA and partial nephrectomy in matched contemporaneous cohorts (97.2% vs 100%; p= 0.31) (10).

During the last few years percutaneous cryoablation has been gaining over thermal ablative techniques as a curative option for small renal cancers. Renal cryoablation was established as a treatment option for renal cancers even before percutaneous RFA as part of open or laparoscopic surgery. With the introduction of thin cryoprobes percutaneous approach was made feasible.

Cryoablation seems to offer some advantages over RFA and other thermal ablation techniques for renal masses. Imaging guidance (CT) allows direct visualization of the ice ball, permitting more precise monitoring of the ablation zone (6). It also allows the simultaneous use and synergy of more than one probe, thus sculpting the ice-ball. Additionally investigators showed that cryoablation has a reduced risk of thermal injury to the collecting system for centrally located tumors (11). This was also recently confirmed by Rosenberg et al. in a series of 41 patients with ice balls overlapping the renal sinus by 6 mm or more (12). A meta-analysis of reported cryoablation vs RFA for small renal masses was published by Kunkle and Uzzo in October 2007 (5). They analyzed the results from forty-seven studies including 1375 renal masses. The meta-analysis demonstrated that repeat ablation was performed more often after RFA (8.5% vs. 1.5%) and the rates of local tumor progression (which includes initial subtotal treatment and late local recurrence) were significantly higher for RFA compared with cryoablation, 12.9% vs. 5.2%, respectively. Atwell et al. in 2012 studied 445 tumors measuring 3.0 cm or smaller treated with thermal ablation (256 tumors were treated with RFA and 189 tumors were treated with cryoablation). They suggested that the two methods are equally effective, having similar major complications and technical success, although cryoablation may be more efficacious for central tumors near the renal hilum (13).

Different studies compared the efficacy of percutaneous and laparoscopic approaches for cryoablation. An analysis of the literature on renal cryoablation from 1966 to 2010 in which 28 laparoscopic studies were compared with 14 percutaneous studies (in total, 1447 tumors) did not show a significant difference in terms of rate of residual tumors (p = 0.25) or rate of recurrent tumor (p = 0.44). The patient groups were comparable in terms of age, tumor size, and duration of follow-up (14).

There are several retrospective studies supporting the short and midterm outcome and efficacy of percutaneous renal cryoablation. Atwell et al. retrospectively reviewed 93 tumors treated with percutaneous cryoablation, with a mean size of 34 mm. They reported technical success rate of 96% with local tumor control in 95% of tumors and 1 case of local tumor progression seen on follow-up (15). In previous studies with smaller series of patients other authors reported local control rates ranging from 83% - 95% based on short term follow up (16, 17, 18, 19). In a prospective study, Buy et al. reviewed 120 tumors with a mean size of 26 mm. They reported a technical success rate of 94% with two tumors requiring second session of cryoablation (either due to recurrence or residual tumor) with disease free survival rate at 1 year of 96.7% (20).

More recently Georgiades et al. published the results of a long-term, prospective study reporting efficacy and safety of percutaneous cryoablation for 265 stage 1A/B renal cancers treated over a period of 5 years. The 5-year cancer specific survival was 100% and the 5-year recurrence free survival was 97%. He reported also an overall significant complication rate of 6%, lower than that of other surgical options, with the most frequent being transfusion-requiring hemorrhage at 1.6% (21). The patients in this study were not limited to those with contraindication to surgery. These results are comparable to the gold-standard (partial nephrectomy) in terms of efficacy, and better in terms of safety.

## Physics of Cryoablation

**Figure 1.**
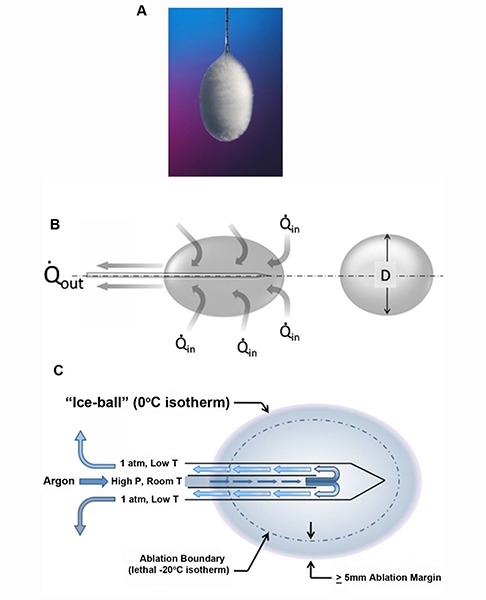
The physics of cryoablation. *Ex-vivo* appearance of the formed ice-ball on a Cryoprobe **(a)**. In human tissue the margin of the ice-ball represents the zero degree (Celsius) isotherm, which is not lethal. As the argon gas drops in pressure it cools substantially and absorbs energy (Q), which is carried away by the warmed gas and released in the room (inert gas). D is the diameter of the visible ice ball **(b)**. The pressurized argon is released inside the probe (no gas is released in the patient). As the pressure drops it cools forming the ice-ball. The lethal ablation zone is 3-5 mm inside the visible ice-ball **(c)**.

**Figure 2.**
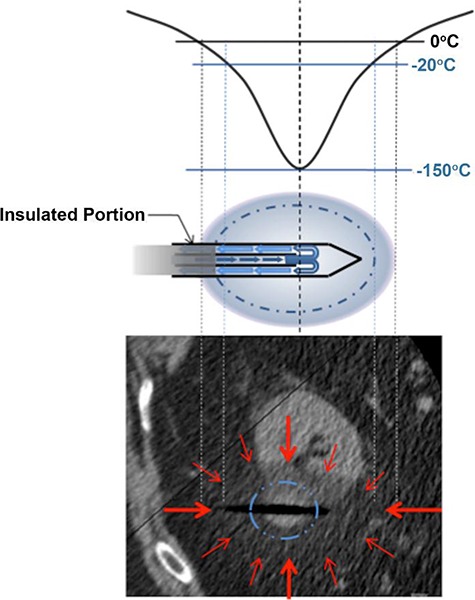
Schematic representation of the relevant isotherms during renal cryoablation. The temperature precipitously increases with distance from the probe. The temperature plot is shown in the upper portion of the figure. The 0^o^ C isotherm is visible and represents the margin of the ‘‘ice-ball,’’ which is not lethal. Lethal temperature for renal tissue is -20-25^o^ C. This isotherm is not visible and resides at a certain distance within the visible ‘‘ice-ball.’’ For effective cryoablation, the nonvisible lethal isotherm must cover the entire target lesion (arrowhead). Note the ‘‘ghost’’ (dark line spearing the target lesion) of the removed cryoprobe as the tissue is still frozen and not collapsed.

**Figure 3.**
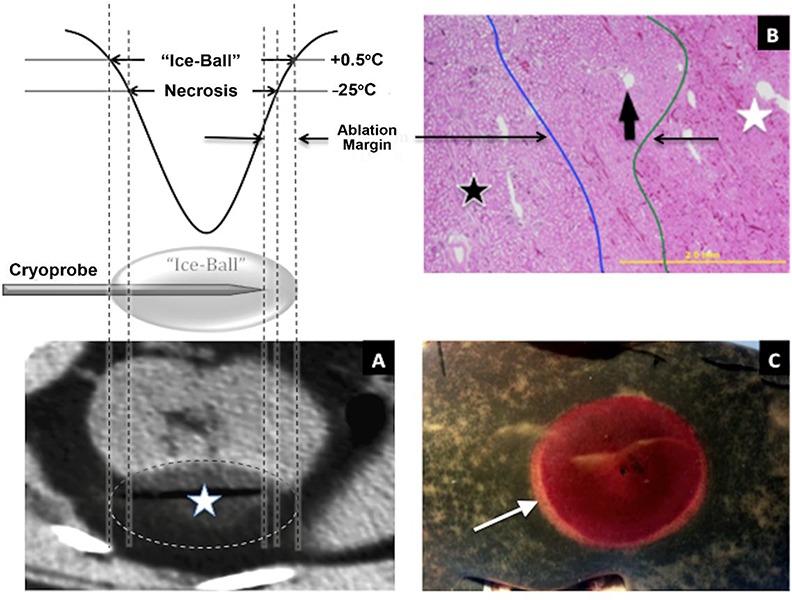
Determination of the lethal cryoablation zone during an animal experiment. The ice-ball is indicated by the dashed line on the CT **(A)**, the blue line on the histopathological slide **(B)** and the arrow on the gross specimen **(C)**. The lethal ablation zone is smaller than the ice-ball and indicated by the green line on the histopathological slide **(B)**. On the gross specimen (c), the lethal zone appears as a red circle inside the ice-ball. In this experiment, the distance between the visible ice- ball and the lethal ablation zone was determined to be about 3 mm (23).

**Figure 4.**
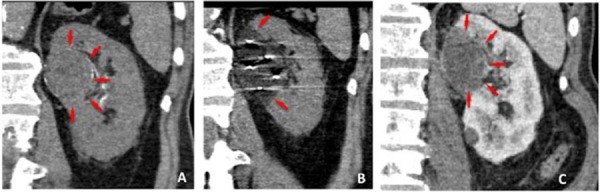
Coronal reformatted, CT of the left kidney **(A**) showing a central biopsy proven renal cell carcinoma (arrows). The intra-procedural coronal image shows the 5 cryyoprobes resulting in a lower density ice-ball (**B,** arrows). Three month follow up CT **(C)** shows complete lack of enhancement of the necrotic tumor (arrows).

Pressurized argon (or other gases that obey the Joules-Thomson law) flows through the double-tubed cryoprobe and as it expands (still inside the probe), cools **(Figure 1)**. Temperature below −20°C has been shown to be sufficient for complete destruction of normal renal parenchyma. Though not substantiated, many authors believe that neoplastic cells are more cryoresistant and may require temperatures as low as −40°C to ensure cell death. Preclinical models demonstrated that lethal temperatures (the ablation zone) can only be achieved within a core volume of tissue at a minimum distance of 3 mm from the edge of the visible ice ball (22, 23) **(Figures 1, 2 and 3)**. Multiple cryoprobes can be used simultaneously. They act synergistically resulting in the formation of even bigger ice ball that can encompass larger tumors. Probes should be positioned 1 cm from the tumor margin and 2 cm from each other **(Figure 4)**.

Cell death during cryoablation is multifactorial. The freezing-thawing cycle that results in both intra and extracellular ice crystal formation, leading to cellular membrane disruption and cell death. Additionally, vessel thrombosis results in ischemic death and finally, low temperatures initiate the process of apoptosis (24).

## Patient selection

Before proceeding to cryoablation all patients should undergo a multiphasic CT or MRI scan. Pre-procedural imaging evaluation is necessary to ensure the patient is a candidate for curative intervention. In general, patients with stage 1A or B are candidates for curative cryoablation. Cross-sectional imaging also allows assessment of the size and location of the mass so that the interventional radiologist can plan the procedure, including number of probes, trajectory and possible risk factors. Vital structures in close proximity to the tumor such as the bowel, can be displaced using water, carbon dioxide or balloons. Placement of a temporary ureteral catheter before the procedure with or without continuous infusion with warm saline can minimize the risk of injury to the ureter. The procedure can be performed under conscious sedation. Biopsy should be performed either before or at the time of ablation to provide information for further management of the patient.

## Follow up

**Figure 5.**
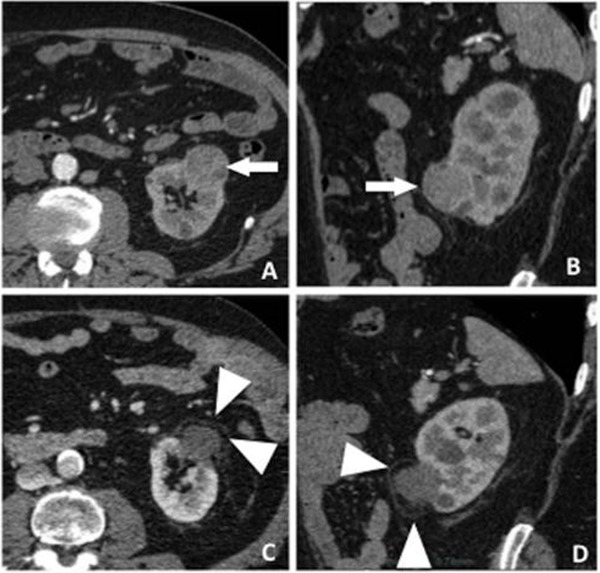
Contrast enhanced, CT scan before cryoablation with axial **(A**) and coronal **(B)** reformations show T1a stage exophytic tumor (white arrows). Contrast enhanced, CT scan at 9 months post-cryoablation with axial **(C)** and coronal **(D**) reformations show no mass enhancement, confirming complete necrosis of the tumor. The inflammatory rim (white arrowheads) is noted representing the edge of the ablation zone and not residual disease.

Although there is no widely accepted post-ablation imaging surveillance protocol to date, imaging at 3-, 6-, 9-, and 12-month post-ablation with contrast-enhanced CT or MRI and at 12-month intervals thereafter, is more than adequate (25). The best indicator of complete ablation is lack of enhancement of a previously enhancing mass **(Figure 5)**. Persistent, ring-type enhancement at the edge of the ablation zone can be seen in most cases and it does not represent residual tumor, rather inflammatory response at the ice-ball margin (26). Nodular or tumor enhancement, on the other hand should raise concern for incomplete treatment or disease recurrence.

## Renal Healing and Function

Since the ablated tissue is not removed, healing is effected by absorption of the dead tissue over time. Over a period of months the dead tissue involutes, shrinks and is eventually replaced by scar tissue. Viability of the tumor on follow up imaging relies in contrast enhancement. If there is complete lack of contrast enhancement of a previously enhancing mass, this is a sign of complete necrosis. Many studies have shown that renal function is not affected by percutaneous ablation. Both creatinine and renal clearance remain stable and the need for dialysis is extremely rare, limited to patients with advanced renal disease and nearly always temporary (17, 21, 27).

## Results and Conclusion

The most rigorous study on the safety and efficacy of image-guided, percutaneous cryoablation for RCC was published by Georgiades et al. (21). The authors treated 134 patients with biopsy proven RCC with cryoablation under CT guidance. Patients included those with stage 1A and B. The 1-, 2-, 3-, 4-, and 5-year efficacy of percutaneous cryoablation for RCC was 99.2, 99.2, 98.9, 98.5, and 97.0%, respectively. Median tumor size was 2.8 ± 1.4 cm. All-cause mortality during the study period was 3 (none from RCC), yielding an overall 5-year survival of 97.8%. The cancer-specific 5-year survival was 100%. No patient developed metastatic disease during the follow-up period. The overall significant Common Terminology Agreement for Adverse Events (CTCAE) version 4.0 complication rate was 6%, with the most frequent being transfusion-requiring hemorrhage, at 1.6%. There was one 30-day-mortality unrelated to the procedure.

Percutaneous ablation of renal tumors under imaging guidance is now a widely accepted nephron sparing curative treatment option for patients who are poor surgical candidates or patients who wish to avoid surgery. These recommendations are supported by prospective, long-term studies (5-year) (21). Treatment failure and local recurrence are uncommon, comparable to that of partial-nephrectomy and do not preclude repeat treatment. In addition, cryoablation appears to be safer than any surgical option. Based on these data, patients with tumors that are stage 1A or B amenable to percutaneous cryoablation, should be offered the option of percutaneous, image guided cryoablation (or thermal ablation).

## References

[1] Homma Y, Kawabe K, Kitamura T, Nishimura Y, Shinohara M, Kondo Y, Saito I, Minowada S, Asakage Y. (1995). Increased incidental detection and reduced mortality in renal cancer: recent retrospective analysis at eight institutions.. Int J Urol.

[2] Fergany AF, Hafez KS, Novick AC. (2000). Long-term results of nephron sparing surgery for localized renal cell carcinoma: 10-year follow-up.. J Urol.

[3] Woldu SL, epub ahead of print (March 2015). Comparison of Renal Parenchymal Volume Preservation Between Partial Nephrectomy, Cryoablation, and Radiofrequency Ablation.. J Endourol.

[4] Campbell SC (2009). Guidelines for management of the clinical T1 renal mass.. J Urol.

[5] Kunkle DA, Uzzo RG. (2008). Cryoablation or radiofrequency ablation of the small renal mass: a meta-analysis.. Cancer.

[6] Venkatesan AM, Wood BJ, Gervais DA. (2011). Percutaneous Ablation in the Kidney.. Radiology.

[7] Breen DJ, Rutherford EE, Stedman B, Roy-Choudhury SH, Cast JE, Hayes MC, Smart CJ. (2007). Management of renal tumors by image-guided radiofrequency ablation: experience in 105 tumors.. Cardiovasc Intervent Radiol.

[8] Zagoria RJ, Traver MA, Werle DM, Perini M, Hayasaka S, Clark PE. (2007 Aug). Oncologic efficacy of CT-guided percutaneous radiofrequency ablation of renal cell carcinomas.. AJR Am J Roentgenol.

[9] Psutka SP, Feldman AS, McDougal WS, McGovern FJ, Mueller P, Gervais DA. (2013). Long-Term Oncologic Outcomes After Radiofrequency Ablation for T1 Renal Cell Carcinoma.. Eur Urol.

[10] Olweny EO, Park SK, Tan YK, Best SL, Trimmer C, Cadeddu JA. (2012). Radiofrequency ablation versus partial nephrectomy in patients with solitary clinical T1a renal cell carcinoma: comparable oncologic outcomes at a minimum of 5 years of follow-up.. Eur Urol.

[11] Janzen NK, Perry KT, Han KR, Kristo B, Raman S, Said JW, Belldegrun AS, Schulam PG. (2005). The effects of intentional cryoablation and radio frequency ablation of renal tissue involving the collecting system in a porcine model.. J Urol.

[12] Rosenberg MD, Kim CY, Tsivian M, Suberlak MN, Sopko DR, Polascik TJ, Nelson RC. (2011). Percutaneous cryoablation of renal lesions with radiographic ice ball involvement of the renal sinus: analysis of hemorrhagic and collecting system complications.. AJR Am J Roentgenol.

[13] Atwell TD (2013). Percutaneous ablation of renal masses measuring 3.0 cm and smaller: comparative local control and complications after radiofrequency ablation and cryoablation.. AJR Am J Roentgenol.

[14] Long CJ, Kutikov A, Canter DJ, Egleston BL, Chen DY, Viterbo R, Boorjian SA, Uzzo RG. (2011). Percutaneous vs surgical cryoablation of the small renal mass: is efficacy compromised?. BJU Int.

[15] Atwell TD, Callstrom MR, Farrell MA, Schmit GD, Woodrum DA, Leibovich BC, Chow GK, Patterson DE, Blute ML, Charboneau JW. (2010). Percutaneous renal cryoablation: local control at mean 26 months of follow up.. J Urol.

[16] Silverman SG, Tuncali K, van Sonnenberg E, Morrison PR, Shankar S, Ramaiya N, Richie JP. (2005). Renal tumors: MR imaging-guided percutaneous cryotherapy—initial experience in 23 patients.. Radiology.

[17] Rodriguez R, Cizman Z, Hong K, Koliatsos A, Georgiades C. (2011). Prospective analysis of the safety and efficacy of percutaneous cryoablation for pT-1NxMx biopsy-proven renal cell carcinoma.. Cardiovasc Intervent Radiol.

[18] Littrup PJ (2007). CT-guided percutaneous cryotherapy of renal masses.. J Vasc Interv Radiol.

[19] Atwell TD, Farrell MA, Leibovich BC, Callstrom MR, Chow GK, Blute ML, Charboneau JW. (2008). Percutaneous renal cryoablation: experience treating 115 tumors.. J Urol.

[20] Buy X1, Lang H, Garnon J, Sauleau E, Roy C, Gangi A. (2013). Percutaneous Renal Cryoablation: Prospective Experience Treating 120 Consecutive Tumors.. AJR Am J Roentgenol.

[21] Georgiades CS, Rodriguez R. (2014). Efficacy and safety of percutaneous cryoablation for stage 1A/B renal cell carcinoma: results of a prospective, single-arm, 5-year study.. Cardiovasc Intervent Radiol.

[22] Campbell SC, Krishnamurthi V, Chow G, Hale J, Myles J, Novick AC. (1998). Renal cryosurgery: experimental evaluation of treatment parameters.. Urology.

[23] Georgiades CS, Rodriguez R, Azene E, Weiss C, Chaux A, Gonzalez-Roibon N, Netto G. (2013). Determination of the Nonlethal Margin Inside the Visible “Ice-Ball” During Percutaneous Cryoablation of Renal Tissue.. Cardiovasc Intervent Radiol.

[24] Clarke DM, Robilotto AT, Rhee E, VanBuskirk RG, Baust JG, Gage AA, Baust JM. (2007). Cryoablation of renal cancer: variables involved in freezing-induced cell death.. Technol Cancer Res Treat.

[25] Allen BC, Remer EM. (2010). Percutaneous Cryoablation of Renal Tumors: Patient Selection, Technique, and Postprocedural Imaging.. Radiographics.

[26] Beemster P, Phoa S, Wijkstra H, de la Rosette J, Laguna P. (2008). Follow-up of renal masses after cryosurgery using computed tomography: enhancement patterns and cryolesion size.. BJU Int.

[27] Kapoor A, Wang Y, Dishan B, Pautler SE. (2014). Update on cryoablation for treatment of small renal mass: oncologic control, renal function preservation, and rate of complications.. Curr Urol Rep.

